# Supplementary material for: An Iron Refractory Phenotype in Obese Adipose Tissue Macrophages Leads to Adipocyte Iron Overload
Source: Int J Mol Sci. 2022 Jul 3;23(13):7417. doi: 10.3390/ijms23137417 (PMC9267114; doi:10.3390/ijms23137417)
Supplement: Supplementary file 1 [file ijms-23-07417-s001.zip › ijms-1787777-supplementary.pdf]

Supplementary Materials

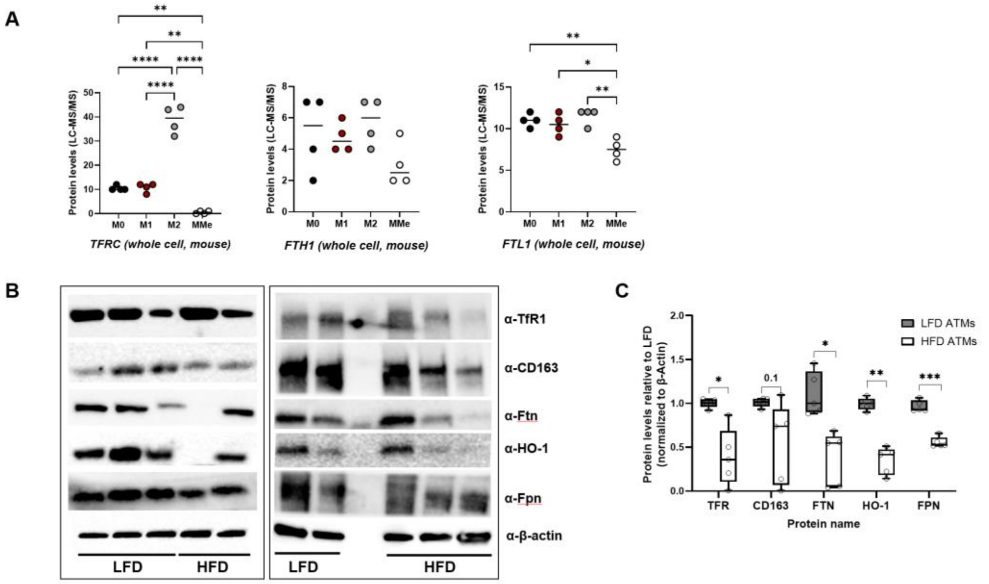

Figure S1. Loss of iron trafficking proteins in bone marrow derived macrophages (BMDMs) and in murine obese adipose tissue macrophages (ATMs). A) Protein levels in polarized BMDMs by liquid chromatography – mass spectrometry (n=4). B-C) Western blot analysis of the expression of iron trafficking proteins in adipose tissue macrophages from mice fed on low-fat (LFD) and high-fat diet (HFD) for 16 weeks and their quantification (n=5). One-way ANOVA with Tukey's multiple comparison *post-hoc* tests were used for data in Panel A and Students *t*-tests were used for data in Panel C. All data are presented as mean ± SEM. *p<0.05, ** p< 0.01, *** p< 0.001, **** p<0.0001.

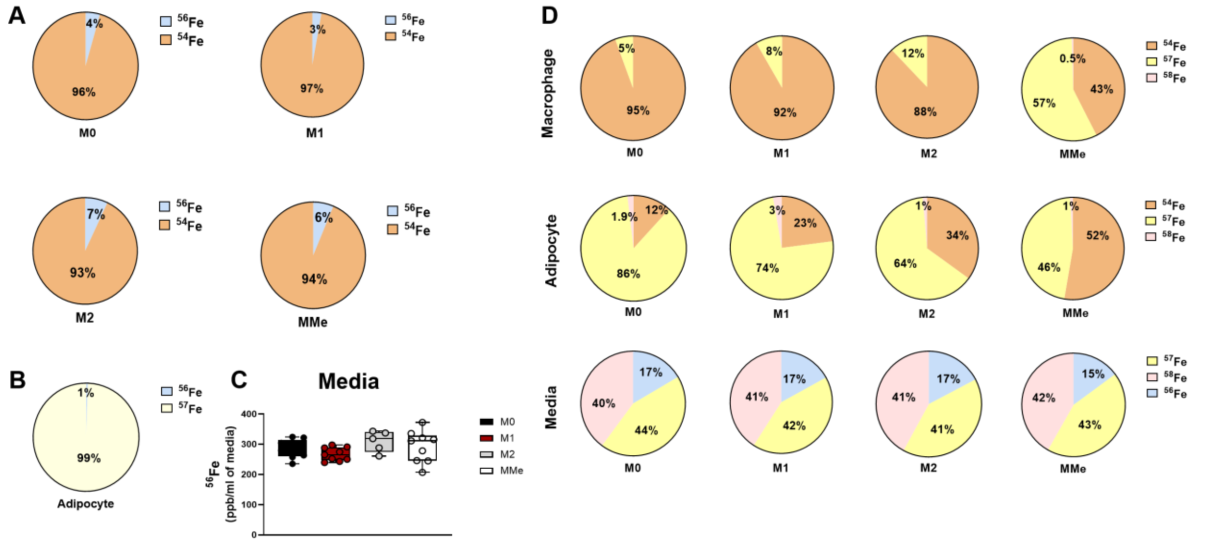

Figure S2. Concentrations of iron isotopes in macrophage and adipocyte cultures. Primary bone marrow derived macrophages (BMDMs) were differentiated and polarized in ^{54}Fe media for up to 8 days while primary adipocytes were differentiated in ^{57}Fe media for 7 days. Concentrations of iron isotopes in A) macrophages B) adipocytes and C) media before co-culture as measured by inductively coupled plasma mass spectrometry. D) Labeled BMDMs and adipocytes were co-cultured in media with residual ^{56}Fe and added ^{58}Fe for 48 h. Concentrations of iron isotopes in macrophages, adipocytes, and media after 48 hours of co-culture were measured by inductively coupled plasma mass spectrometry.

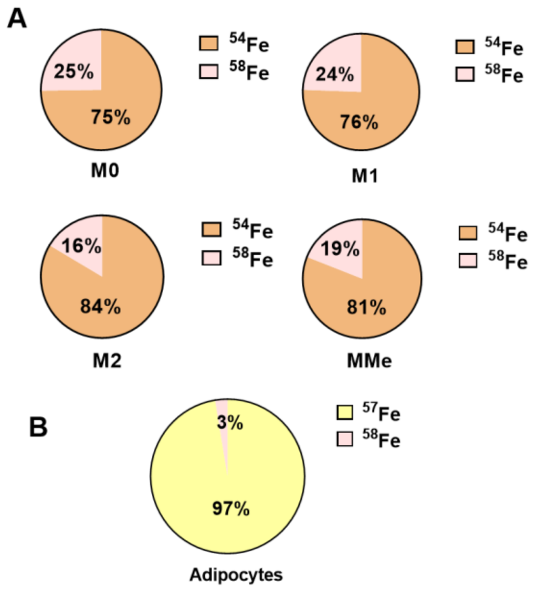

Figure S3. Iron remaining in macrophages and adipocytes after individual culture in media with ^{58}Fe. Primary bone marrow derived macrophages were differentiated and polarized in ^{54}Fe media for up to 8 days. Primary adipocytes were differentiated in ^{57}Fe media for 7 days. Instead of being co-cultured in ^{58}Fe containing media, they were cultured individually for 48 h followed by iron isotope quantification using inductively coupled plasma mass spectrometry.

Table S1: Primers and antibodies used in this study

**Gene name**	**Assay ID**	**Supplier**	**Assay**	**Citations**
Transferrin receptor (Tfrc)	Mm00441941_m1	Thermo Fisher/TaqMan	RT-qPCR	>50
Cd163	Mm00474091_m1	Thermo Fisher/TaqMan	RT-qPCR	>20
Cd91 (Lrp1)	Mm00464608_m1	Thermo Fisher/TaqMan	RT-qPCR	20
Ferritin light chain 1 (Ftl1)	Mm03030144_g1	Thermo Fisher/TaqMan	RT-qPCR	10
Ferritin heavy chain 1 (Fth1)	Mm00850707_g1	Thermo Fisher/TaqMan	RT-qPCR	>10
Heme oxygenase 1 (Hmox1)	Mm00516005_m1	Thermo Fisher/TaqMan	RT-qPCR	>100
Ferroportin (Slc40a1)	Mm01254822_m1	Thermo Fisher/TaqMan	RT-qPCR	9
Itgax	Mm00498698_m1	Thermo Fisher/TaqMan	RT-qPCR	>45
Tnfa	Mm00443258_m1	Thermo Fisher/TaqMan	RT-qPCR	>1200
Mrc1	Mm01329359_m1	Thermo Fisher/TaqMan	RT-qPCR	2
Egr2	Mm00456650_m1	Thermo Fisher/TaqMan	RT-qPCR	>25
Abca1	Mm00442646_m1	Thermo Fisher/TaqMan	RT-qPCR	55
Plin2	Mm00475794_m1	Thermo Fisher/TaqMan	RT-qPCR	>10
B2M	Mm00437762_m1	Thermo Fisher/TaqMan	RT-qPCR	>315
Gapdh	Mm99999915_g1	Thermo Fisher/TaqMan	RT-qPCR	>2820
Actb	Mm00607939_s1	Thermo Fisher/TaqMan	RT-qPCR	>940
**Antibody name**	**Catalog number**	**Supplier**	**Assay**	**Dilution**
Transferrin receptor	13-6800	Thermo Fisher	WB	1:1000
CD163	93498S, ab182422	Cell Signaling, Abcam	WB	1:250
Ferritin light chain 1	ab69090	Abcam	WB	1:1000
Ferritin heavy chain 1	ab75972	Abcam	WB	1:1000
Ferritin	ab75973	Abcam	WB	1:1000
Heme oxygenase	ab13243	Abcam	WB	1:1000
Ferroportin (Slc40a1)	NBP1-215022	Novus Bio	WB	1:1000
β-actin	3700S	Cell Signaling	WB	1:1000
Fc Block	553141	BD Biosciences	FACS	1:100
APC F4/80	123116	Biolegend	FACS	1:200
FITC-CD11b	14-0112-82	eBioscience	FACS	1:200
PE-CD45	103106	eBioscience	FACS	1:400
DAPI	D1306	Thermo Fisher	FACS	1:4000
F4/80 beads	130-110-443	Miltenyi	ATM Magnetic sort	1:10
